# Simultaneously characterizing the comparative economics of routine female adolescent nonavalent human papillomavirus (HPV) vaccination and assortativity of sexual mixing in Hong Kong Chinese: a modeling analysis

**DOI:** 10.1186/s12916-018-1118-3

**Published:** 2018-08-17

**Authors:** Horace C. W. Choi, Mark Jit, Gabriel M. Leung, Kwok-Leung Tsui, Joseph T. Wu

**Affiliations:** 1 WHO Collaborating Centre for Infectious Disease Epidemiology and Control, School of Public Health, Li Ka Shing Faculty of Medicine, The University of Hong Kong, 1/F North Wing, Patrick Manson Building, 7 Sassoon Road, Pok Fu Lam, Hong Kong; 2 Department of Systems Engineering and Engineering Management, City University of Hong Kong, Kowloon Tong, Hong Kong; 3 Department of Clinical Oncology, Li Ka Shing Faculty of Medicine, The University of Hong Kong, Pok Fu Lam, Hong Kong; 4 Modelling and Economics Unit, Public Health England, London, UK; 5 Department of Infectious Disease Epidemiology, London School of Hygiene and Tropical Medicine, London, UK

**Keywords:** Human papillomavirus, Vaccination, Cervical cancer, Hong Kong, Cost-benefit analysis, Cost-effectiveness analysis, Mathematical model, Sexual mixing

## Abstract

**Background:**

Although routine vaccination of females before sexual debut against human papillomavirus (HPV) has been found to be cost-effective around the world, its cost-benefit has rarely been examined. We evaluate both the cost-effectiveness and cost-benefit of routine female adolescent nonavalent HPV vaccination in Hong Kong to guide its policy, and by extension that of mainland China, on HPV vaccination. One major obstacle is the lack of data on assortativity of sexual mixing. Such difficulty could be overcome by inferring sexual mixing parameters from HPV epidemiologic data.

**Methods:**

We use an age-structured transmission model coupled with stochastic individual-based simulations to estimate the health and economic impact of routine nonavalent HPV vaccination for girls at age 12 on cervical cancer burden and consider vaccine uptake at 25%, 50%, and 75% with at least 20 years of vaccine protection. Bayesian inference was employed to parameterize the model using local data on HPV prevalence and cervical cancer incidence. We use the human capital approach in the cost-benefit analysis (CBA) and GDP per capita as the indicative willingness-to-pay threshold in the cost-effectiveness analysis (CEA). Finally, we estimate the threshold vaccine cost (TVC), which is the maximum cost for fully vaccinating one girl at which routine female adolescent nonavalent HPV vaccination is cost-beneficial or cost-effective.

**Results:**

As vaccine uptake increased, TVC decreased (i.e., economically more stringent) in the CBA but increased in the CEA. When vaccine uptake was 75% and the vaccine provided only 20 years of protection, the TVC was US$444 ($373–506) and $689 ($646–734) in the CBA and CEA, respectively, increasing by approximately 2–4% if vaccine protection was assumed lifelong. TVC is likely to be far higher when non-cervical diseases are included. The inferred sexual mixing parameters suggest that sexual mixing in Hong Kong is highly assortative by both age and sexual activity level.

**Conclusions:**

Routine HPV vaccination of 12-year-old females is highly likely to be cost-beneficial and cost-effective in Hong Kong. Inference of sexual mixing parameters from epidemiologic data of prevalent sexually transmitted diseases (i.e., HPV, chlamydia, etc.) is a potentially fruitful but largely untapped methodology for understanding sexual behaviors in the population.

**Electronic supplementary material:**

The online version of this article (10.1186/s12916-018-1118-3) contains supplementary material, which is available to authorized users.

## Background

The cost-effectiveness of routine female adolescent human papillomavirus (HPV) vaccination and other strategies (e.g., vaccinating males as well) has been extensively studied for many high-income countries (e.g., the UK, Australia, Canada) as well as middle- and low-income countries (e.g., Malaysia, Brazil, Peru) [1]. The consensus among these studies is that routine female adolescent HPV vaccination is cost-effective. In contrast, very few studies have examined the corresponding cost-benefit, which is an important alternative criterion for health technology assessment because (1) in some jurisdictions, such as Hong Kong, health policymaking is based on cost-benefit instead of cost-effectiveness; (2) economists have suggested that cost-benefit analysis (CBA) is able to capture a wider range of the benefits of vaccination compared to cost-effectiveness analysis (CEA) [2]; and (3) CBA and CEA may lead to discordant conclusions regarding the economic favorability of health interventions due to different but equally sound methodologies and assumptions [3].

Despite the recent advent of a second-generation HPV vaccine (which targets nine HPV types that account for approximately 90% of cervical cancer worldwide [4]), Hong Kong and mainland China have not yet decided whether to include HPV vaccines in their routine immunization programs. The primary objective of this study is to provide a robust evidence base for HPV vaccination policy in the sentinel Chinese population of Hong Kong by performing both CBA and CEA of routine female adolescent nonavalent HPV vaccination for reducing cervical cancer burden using methodology that conforms with health technology assessments in this city. The health technology assessments framework for HPV vaccination in Hong Kong can serve as a reference for mainland China’s public health policy on prevention of cervical cancer, which is the second-most common cancer in women aged below 45 in the country [5].

A key challenge in a rigorous evaluation of HPV vaccination programs is to adequately parameterize the HPV transmission model. To robustly evaluate the health impact of HPV vaccination, evidence-based HPV transmission models are needed to estimate the herd immunity effect [6]. Extensive research in recent years has identified assortativity of sexual mixing and the duration of naturally acquired immunity as two major sources of uncertainty in our understanding of HPV transmission dynamics [7–9]. Regarding sexual mixing, models for heterosexual transmission of HPV require specification on (1) heterogeneity in sexual activity levels (e.g., the number of sexual partners over the past year) within each age group and (2) assortativity of sexual mixing by age and sexual activity level (i.e., how differences in age and sexual activity level between two individuals affect their probability of forming a sexual partnership). Most populations, including Hong Kong, lack empirical data on point (2), in which case assortativity of sexual mixing is modeled by either extrapolating from the few populations with such data (e.g., a study for Austria used the sexual mixing data from the UK [10]) or by making hypothetical assumptions (e.g., [11]). Regarding natural immunity, although a recent meta-analysis suggested that natural recovery from HPV infection provides modest short-term protection against re-infection among females [9], the longevity of this effect remains unknown. To account for the uncertainty in assortativity of sexual mixing and natural immunity, we adopt the novel approach of Korostil et al. [8], who suggested that the underlying parameters could be inferred from HPV epidemiologic data during model parameterization. Consequently, as we evaluate the cost-benefit and cost-effectiveness of routine female adolescent nonavalent HPV vaccination for Hong Kong, we will also be simultaneously characterizing the underlying assortativity of heterosexual mixing, which in itself is an important knowledge gap on sexually transmitted infections.

## Methods

Herein, we briefly describe the model structure. Please see the Additional file 1 for more details on the model.

### Model overview

Our model comprises (1) a deterministic age-structured compartmental dynamic model for simulating heterosexual transmission of high-risk HPV (HR-HPV) and (2) a stochastic individual-based cohort model for simulating the development of cervical cancer over the lifetime of each female. This hybrid approach has been used in previous studies of HPV vaccination (e.g., [11]). We group HR-HPV into four classes: (1) HPV-16; (2) HPV-18; (3) HPV-OV (for ‘other vaccine types’), which comprises the other five HR-HPV targeted by the nonavalent vaccine, namely HPV-31, 33, 45, 52, and 58; and (4) HPV-NV, which comprises all the non-vaccine HR-HPV. The dynamic model is used to estimate the model parameters and the herd immunity effect after routine female adolescent HPV vaccination has begun. The age-specific force of infection from the dynamic model is fed into the stochastic individual-based model to simulate cervical cancer incidence for each birth cohort. The cohort model explicitly simulates cervical screening, which cannot be accurately and easily performed with compartmental models because of the history-dependent nature of screening and treatment per the guidelines issued by the Hong Kong College of Obstetricians and Gynecologists [12]. The time step is 1 month in all simulations. The maximum age is 85 years for all individuals.

### Natural history

Each individual enters the model at age 10 without any HPV infection (Additional file 1: Figure S1). After a female has been infected, she remains free of lesions for some time and then either clears the infection or progresses to cervical intraepithelial neoplasia (CIN1, 2, or 3). We assume that individuals with CIN3 do not recover naturally and will eventually progress to cervical cancer if untreated. The mean duration of natural immunity for HPV-16 and HPV-18 from HPV epidemiologic data are inferred during model parameterization (see below). HPV-OV and HPV-NV each comprise multiple HR-HPV that are unlikely to have significant cross-immunity [13]. As such, we assume no natural immunity for HPV-NV (i.e., individuals remain fully susceptible after clearance of infection). In contrast, to avoid overestimating the herd immunity effect conferred by vaccination [8], we allow natural immunity for HPV-OV and infer its mean duration during model parameterization. We assume that natural immunity provides 100% protection against reinfection of the same HPV class and that its duration is exponentially distributed. We model co-infections among the four HPV classes by assuming that disease progression of a given co-infection follows the progression rate of the most aggressive class therein, whereas class-specific clearance is unaffected by co-infections. We assume that the duration of HPV infection is the same among males and females [11]. When estimating the health burden associated with HPV, we consider only cervical cancer because Hong Kong does not have robust age-specific data on incidence of genital warts (more than 90% of which are caused by HPV-6 and HPV-11, against which the nonavalent vaccine is more than 90% efficacious) and other forms of HPV-associated cancers (e.g., vulvar, penile). As such, our study will tend to underestimate the health and economic benefits of nonavalent HPV vaccination.

### Sexual mixing

We stratify the population into two sexes (*g* ∈ {*f*, *m*}), 76 1-year age groups (*a* = 10,11,12,…,85), and three sexual activity levels (*s* ∈ {*none*, *low*, *high*} that denote no, one, and multiple sexual partners during the past 6 months, respectively). Let *N*_*g*, *a*, *u*_(*t*) be the number of individuals in stratum (*g*, *a*, *u*) at time *t,* and *c*_*g*, *a*, *u*_ be the rate at which these individuals form new sexual partnerships. The age-specific distribution of individuals with different sexual activity levels are based on the sexuality study results published by the Family Planning Association of Hong Kong (FPAHK) [14]; see Additional file 1 for details. We model assortativity of sexual mixing by age and sexual activity based on the formulation in Walker et al. [15]. Specifically, given that an individual in stratum (*g*, *a*, *u*) forms a sexual partnership at time *t*, the probability that their partner belongs to stratum (*g'*, *b*, *v*),  *g* ≠ *g'*, is

$$ {\displaystyle \begin{array}{l}{\rho}_{g,a,u,b,v}(t)={\varepsilon}_A{\varepsilon}_S\underset{\begin{array}{c}\mathrm{assortative}\ \mathrm{mixing}\ \mathrm{for}\ \mathrm{both}\ \\ {}\mathrm{age}\ \mathrm{and}\ \mathrm{sexual}\ \mathrm{activity}\ \mathrm{level}\end{array}}{\underbrace{\Phi \left(\frac{a-b}{\sigma_g}\right){\delta}_{uv}}}+{\varepsilon}_A\left(1-{\varepsilon}_S\right)\underset{\begin{array}{c}\mathrm{assortative}\ \mathrm{mixing}\ \mathrm{for}\ \mathrm{age}\\ {}\ \mathrm{proportionate}\ \mathrm{mixing}\ \mathrm{for}\ \mathrm{sexual}\ \mathrm{activity}\ \mathrm{level}\end{array}}{\underbrace{\Phi \left(\frac{a-b}{\sigma_g}\right)\frac{c_{g^{\prime },b,v}{N}_{g^{\prime },b,v}(t)}{\sum \limits_l{c}_{g^{\prime },b,l}{N}_{g^{\prime },b,l}(t)}}}\\ {}+\left(1-{\varepsilon}_A\right){\varepsilon}_S\underset{\begin{array}{c}\mathrm{proportionate}\ \mathrm{mixing}\ \mathrm{for}\ \mathrm{age}\\ {}\ \mathrm{assortative}\ \mathrm{mixing}\ \mathrm{for}\ \mathrm{sexual}\ \mathrm{activity}\ \mathrm{level}\end{array}}{\underbrace{\frac{c_{g^{\prime },b,v}{N}_{g^{\prime },b,v}(t)}{\sum \limits_k{c}_{g^{\prime },k,v}{N}_{g^{\prime },k,v}(t)}{\delta}_{uv}}}+\left(1-{\varepsilon}_A\right)\left(1-{\varepsilon}_S\right)\underset{\begin{array}{c}\mathrm{proportionate}\ \mathrm{mixing}\ \mathrm{for}\ \mathrm{both}\\ {}\mathrm{age}\ \mathrm{and}\ \mathrm{sexual}\ \mathrm{activity}\ \mathrm{level}\end{array}}{\underbrace{\frac{c_{g^{\prime },b,v}{N}_{g^{\prime },b,v}(t)}{\sum \limits_k{\sum}_l{c}_{g^{\prime },k,l}{N}_{g^{\prime },k,l}(t)}}}\end{array}} $$

where *δ*_*uv*_ has value 1 when *u* = *v* and 0 otherwise, and Φ(⋅) is the Gaussian kernel. We use the Gaussian kernel to model age assortativity because its shape conforms with intuition as well as the patterns empirically observed in sexual activity surveys from the UK, Australia, and the US [16–18]. In this formulation, age assortativity is controlled by *ε*_*A*_ and *σ*_*g*_ whereas risk assortativity is controlled by *ε*_*S*_. For simplicity, we assume that *σ*_*g*_ is the same for males and females.

Let *I*_*g*, *a*, *u*, *h*_(*t*) be the prevalence of HPV class *h* among individuals in stratum (*g*, *a*, *u*) at time *t*. The force of infection from HPV class *h* for individuals in stratum (*g*, *a*, *u*) at time *t* is

$$ {\lambda}_{g,a,u,h}(t)=\sum \limits_b\sum \limits_v\left[{\alpha}_a{\alpha}_b{\beta}_h{c}_{g,a,u,b,v}^{\ast }(t){\rho}_{g,a,u,b,v}(t)\frac{I_{g^{\prime },b,v,h}(t)}{N_{g^{\prime },b,v}(t)}\right] $$

where *β*_*h*_ is the class-specific baseline probability of transmission per sexual partnership, ${c}_{g,a,u,b,v}^{\ast }(t)$ is the adjusted contact rate between stratum (*g*, *a*, *u*) and (*g'*, *b*, *v*) (see Additional file 1 for details), and

$$ {\alpha}_a=\left\{\begin{array}{l}1\\ {}1+\frac{\mu -1}{W_2-{W}_1}\left(a-{W}_1\right)\\ {}\mu \end{array}\kern0.5em \begin{array}{l}\mathrm{if}\ a<{W}_1\\ {}\mathrm{if}\ {W}_1\le a\le {W}_2\\ {}\mathrm{if}\ a>{W}_2\end{array}\right. $$

for modeling the effect of age on susceptibility and infectiousness (e.g., to reflect the age dependence of condom use, which increased from 26% in the 15–19 age group to 70% in the 18–27 age group according to the Youth Sexuality Study 2011 by the FPAHK [19]). The model assumes that susceptibility and infectiousness is (1) highest (*α*_*a*_ = 1) for individuals aged below *W*_1_; (2) linearly decreases from 1 to *μ* as individuals age from *W*_1_ to *W*_2_; and (3) remains at *α*_*a*_ = *μ* for individuals aged above *W*_2_. The parameters *μ*, *W*_1_, and *W*_2_ are inferred during model parameterization.

### Model parameterization

The following epidemiologic data were used to parameterize the model:

1. Age-specific prevalence of HR-HPV in Hong Kong as reported in two previous studies [20, 21].
2. Age-specific cervical cancer incidence in 1980–1984 as recorded by the Hong Kong Cancer Registry [22]. We choose this period to minimize the confounding effect of screening on cervical cancer incidence (there are no data on screening coverage in Hong Kong before 2000).
3. HR-HPV distribution among cervical cancer cases in Hong Kong hospitals during 1972–1973 and 1984–1986 [23].
4. The cumulative proportion of cases with disease progression and recovery for different stages of HPV infection from two overseas studies [24, 25]. Analogous data are not available in Hong Kong.

We infer the following model parameters by fitting the model to these data using Markov chain Monte Carlo methods with non-informative flat priors [7]: (1) class-specific progression and clearance rates for different stages of HPV infection; (2) the mean duration of natural immunity for HPV-16, HPV-18, and HPV-OV; (3) baseline class-specific transmission probability per sexual partnership (*β*_*h*_); (4) assortativity of sexual mixing (*ε*_*A*_, *σ*_*g*_, and *ε*_*S*_); and (5) age-specific susceptibility and infectiousness (*μ*, *W*_1_, and *W*_2_).

### Routine HPV vaccination

We compare routine vaccination for girls at age 12 to opportunistic vaccination with status quo vaccine uptake (12% [26]). We assume that the nonavalent HPV vaccines are used in both routine HPV vaccination and status quo opportunistic vaccination and that full vaccination is provided by the two-dose regime recommended for individuals aged 9–14 years in Hong Kong [27]. Our previous survey suggested that approximately 40–50% of mothers in Hong Kong would consent to HPV vaccination for their adolescent daughters [28]. On the other hand, the uptake in the UK and Australia is 70–80%, which is the highest around the world [29, 30]. As such, we consider three scenarios of vaccine uptake for routine vaccination, namely 25%, 50%, and 75%. The class-specific vaccine efficacy is as follows [4, 31]: (1) 95.5% (95% confidence interval 90.0%–98.4%) for HPV-16; (2) 95.8% (84.1%–99.5%) for HPV-18; (3) 96.0% (94.4%–97.2%) for HPV-OV; and (4) 0% for HPV-NV. The latest clinical trial results showed that individuals who were vaccinated at age 9–15 years while they were still sexually naive remained seropositive (against vaccine-type HPV) after 10 years [32]. A modeling study used immunogenicity data to estimate that vaccine protection will likely persist for at least 20 years [33]. As such, we consider three possibilities, namely 20-year, 30-year, and lifelong protection.

### Cervical cancer screening

We assume that vaccination does not affect screening behavior. The Cervical Screening Programme (CSP) in Hong Kong recommends women aged between 25 and 65 years to follow the 1-, 1-, 3-yearly cycle of cervical screening (i.e., screening annually for their first 2 years of screening and then triennially if their screening results remain negative) [27]. Based on the screening uptake data from the Behavioral Risk Factor Surveillance System surveys [27] and published literature [34, 35], we assume that screening uptake increased linearly from 40% in 1980 to 70% in 2004 when CSP was launched and remained at 70% thereafter. We assume that the sensitivity of cervical cytology at the threshold of atypical squamous cells of undetermined significance in detecting CIN2/3 and cervical cancer are 80% and 100%, respectively [36] (see Additional file 1 for details).

### CBA and CEA

In the CBA, we used the human capital approach to monetize health and life-year loss into productivity loss based on average personal income [37] (see Additional file 1 for details). In the CEA, we set the willingness-to-pay threshold for the incremental cost-effectiveness ratio (defined as the additional cost per each quality-adjusted life-year gained when comparing two interventions) at one local gross domestic product (GDP) per capita which is the lowest threshold used in cost-effectiveness studies of vaccination programs for Hong Kong [38]. The average GDP per capita in Hong Kong during 2012–2016 was US$40,099 [39]. To assess the long-term impact of HPV vaccination, we estimated the changes in costs and health across the lifetimes of all female cohorts over a time horizon of 100 years. For example, at year 99, the incoming cohort will incur costs and benefits over its lifetime. The costs of screening and treatments were based on (1) charges for private patients in public hospitals, which account for over 90% of inpatient care in Hong Kong [40], and (2) expert opinions among local oncologists and gynecologists. Health utility parameters were based on overseas studies due to the lack of local data [41, 42]. Following the WHO guidelines, we discounted cost and health utility for women regardless of their ages after year 1 at 3% per annum [43]; the first age that discounting began was age 10 years. All cost figures were denominated in US dollars.

### Cost of vaccination

We set the vaccine cost in the status quo scenario at US$284 based on the price list in the FPAHK (which is a non-profit organization) [14] and the two-dose regime as recommended for individuals aged 9–14 years in Hong Kong [27]. Instead of explicitly modeling vaccine dose schedules and costs for routine HPV vaccination, we considered the cost required to fully vaccinate one girl (which includes the procurement, logistical, and administrative costs) as the outcome of our CBA and CEA. With this approach, we performed a head-to-head comparison between the CBA and CEA threshold vaccine cost, which was defined as the highest cost of vaccination at which the routine HPV vaccination program is cost-beneficial (i.e., the net monetary benefit is positive) and cost-effective (i.e., the incremental cost-effectiveness ratio is below the willingness-to-pay threshold), respectively. We denoted the CBA and CEA threshold vaccine cost by *TVC*_*CBA*_ and *TVC*_*CEA*_, respectively.

### Probabilistic sensitivity analysis

For each vaccination uptake and protection scenario, we considered 10,000 probabilistic sensitivity analysis scenarios which comprise all combinations of (i) 100 sets of transmission and natural history parameters randomly generated from their posterior distributions obtained from model parameterization (Additional file 1: Table S2); and (ii) 100 sets of vaccine efficacy, screening, cost, and health utility parameters randomly generated from their plausible ranges shown in Additional file 1: Tables S3–S5 [4, 41, 42].

## Results

### Transmissibility and duration of natural immunity

**Fig. 1:**
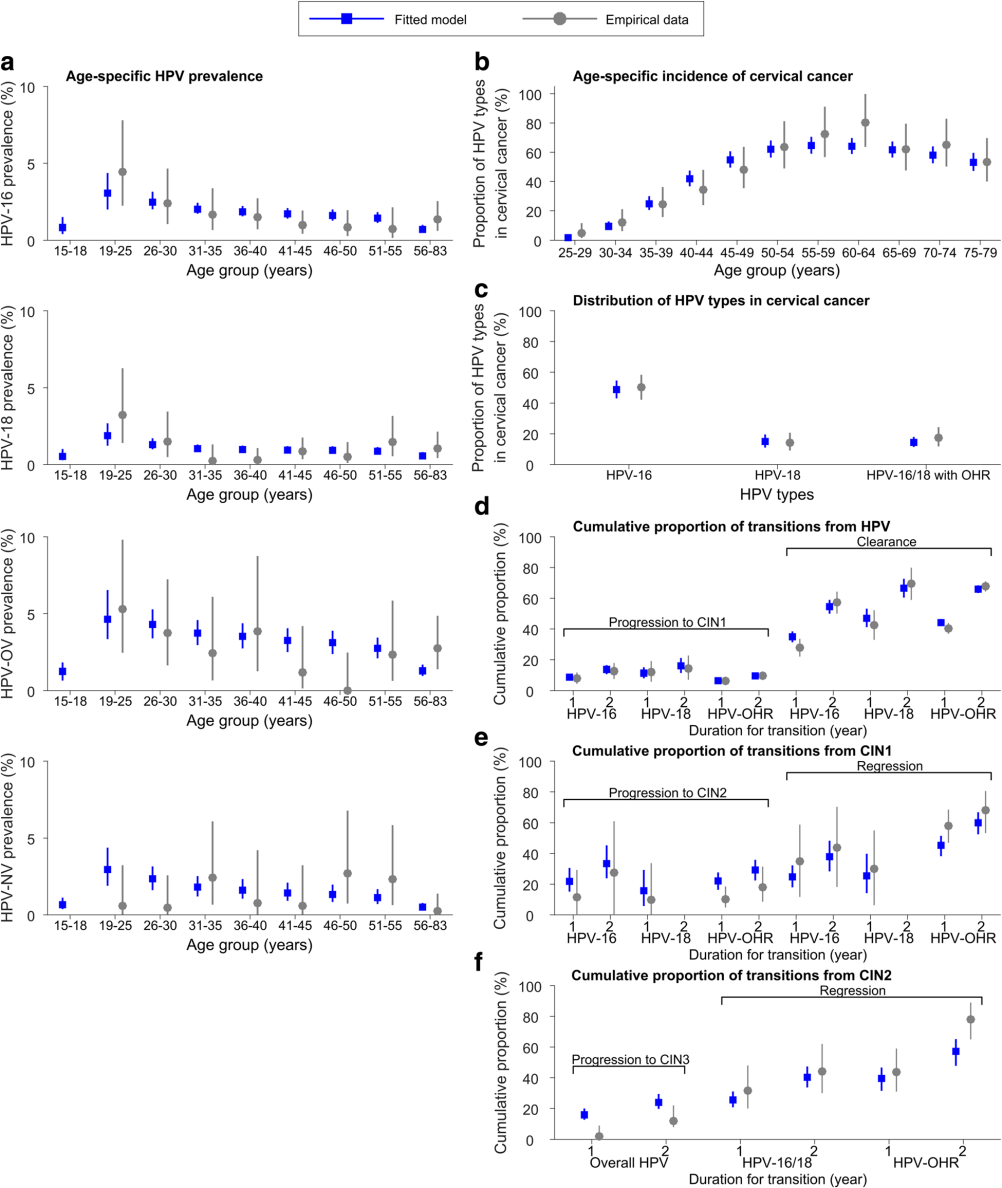
Comparison of the empirical data and the fitted model. **a** Age-specific HPV prevalence. **b** Age-specific incidence of cervical cancer. **c** Distribution of HPV types in cervical cancer lesions. **d**–**f** Cumulative proportions of disease progression and clearance within 2 years for HPV infections without lesions, CIN1 and CIN2. Error bars indicate the 95% credible intervals for the fitted model and 95% confidence intervals for the data

**Fig. 2:**
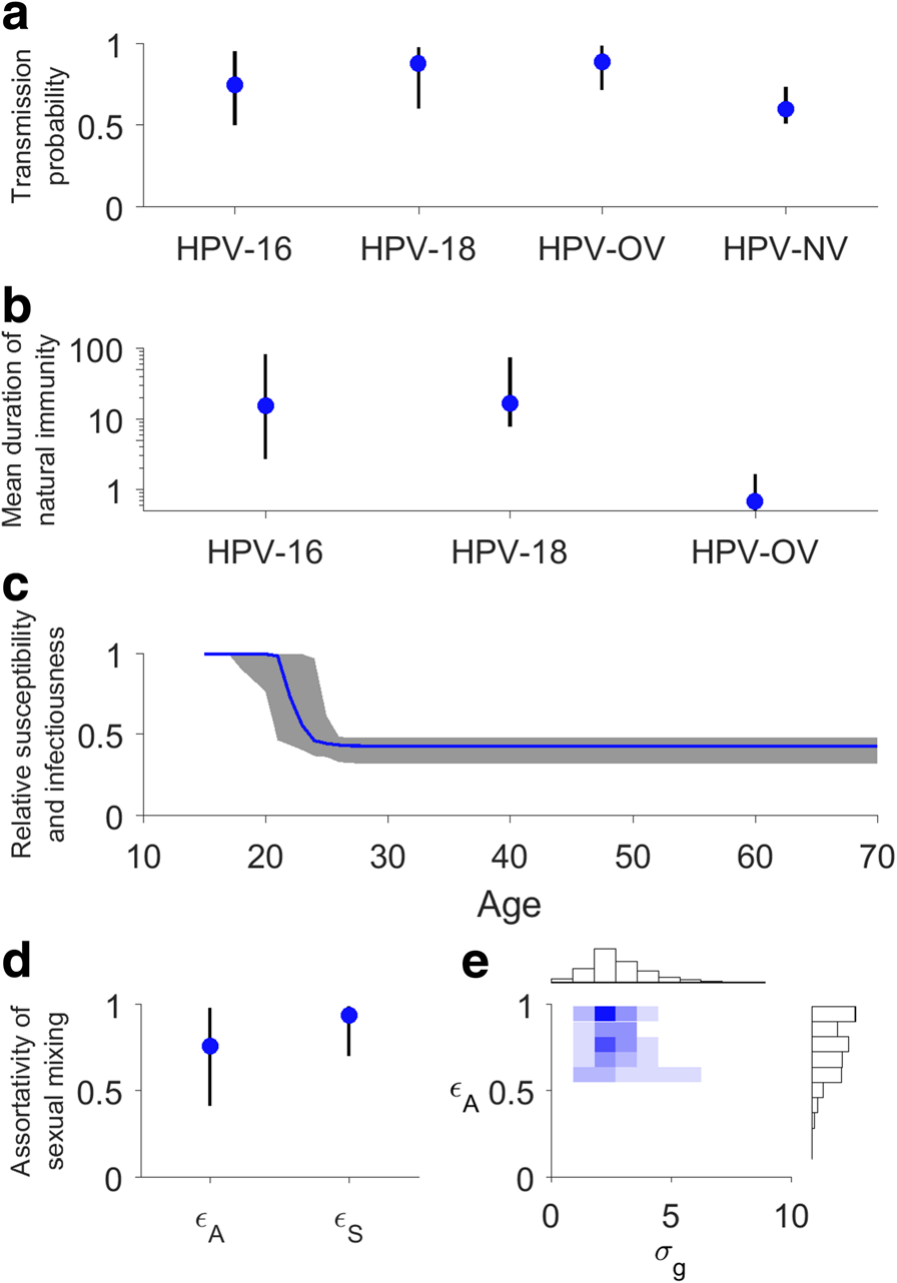
Key parameter estimates in the fitted model. Circles and vertical lines indicate posterior medians and 95% credible intervals. **a** Baseline probability of HPV transmission per sexual partnership. **b** Mean duration of natural immunity (on log-scale). **c** Age-specific susceptibility and infectiousness. The line and shades indicate medians and 95% credible intervals. **d** *ε*_*A*_ and *ε*_*S*_ for assortativity of sexual mixing. **e** Joint posterior distribution of *ε*_*A*_ and *σ*_*g*_. Higher values of *ε*_*A*_ and smaller values of *σ*_*g*_ mean higher degree of age assortativity. Darker color indicates higher density. See Additional file 1: Table S2 for all inferred parameter values

The fitted model was largely congruent with the epidemiologic data used for model parameterization (Fig. 1). The baseline probability of transmission per partnership was 0.75 (95% credible interval 0.50–0.96), 0.88 (0.60–0.98), 0.93 (0.80–0.99), and 0.61 (0.50–0.71) for HPV-16, HPV-18, HPV-OV, and HPV-NV, respectively (Fig. 2a). The mean duration of natural immunity was 16 (3–83), 17 (4–75), and 0.7 (0.5–1.7) years for HPV-16, HPV-18, and HPV-OV, respectively (Fig. 2b). The inferred ephemeralness of natural immunity for HPV-OV was consistent with its multi-type nature (i.e., clearance of one type of HPV-OV will unlikely prevent infection by other types of HPV-OV). As individuals reach age 21 (16–24), their susceptibility and infectiousness began to fall gradually until they reached age 24 (21–27) (Fig. 2c). The total decrease in susceptibility and infectiousness over this period was 53% (47%–59%).

### Assortativity of sexual mixing

Sexual mixing was highly assortative by both age (*ε*_*A*_ = 0.77 (0.29 – 0.99); *σ*_*g*_ = 2.1 (0.2 – 0.49)) and sexual activity level (*ε*_*S*_ = 0.98 (0.89 – 0.99)) (Fig. 2d, e). The inferred level of age assortativity in sexual mixing was comparable with that empirically observed in sexual surveys from the UK [44] and Australia [17]. In our fitted model, 74% (43%–97%) and 84% (60%–99%) of adult heterosexual partnerships had less than 5 and 10 years of age difference between the two partners, respectively.

### Epidemiologic impact of routine female adolescent HPV vaccination

**Fig. 3:**
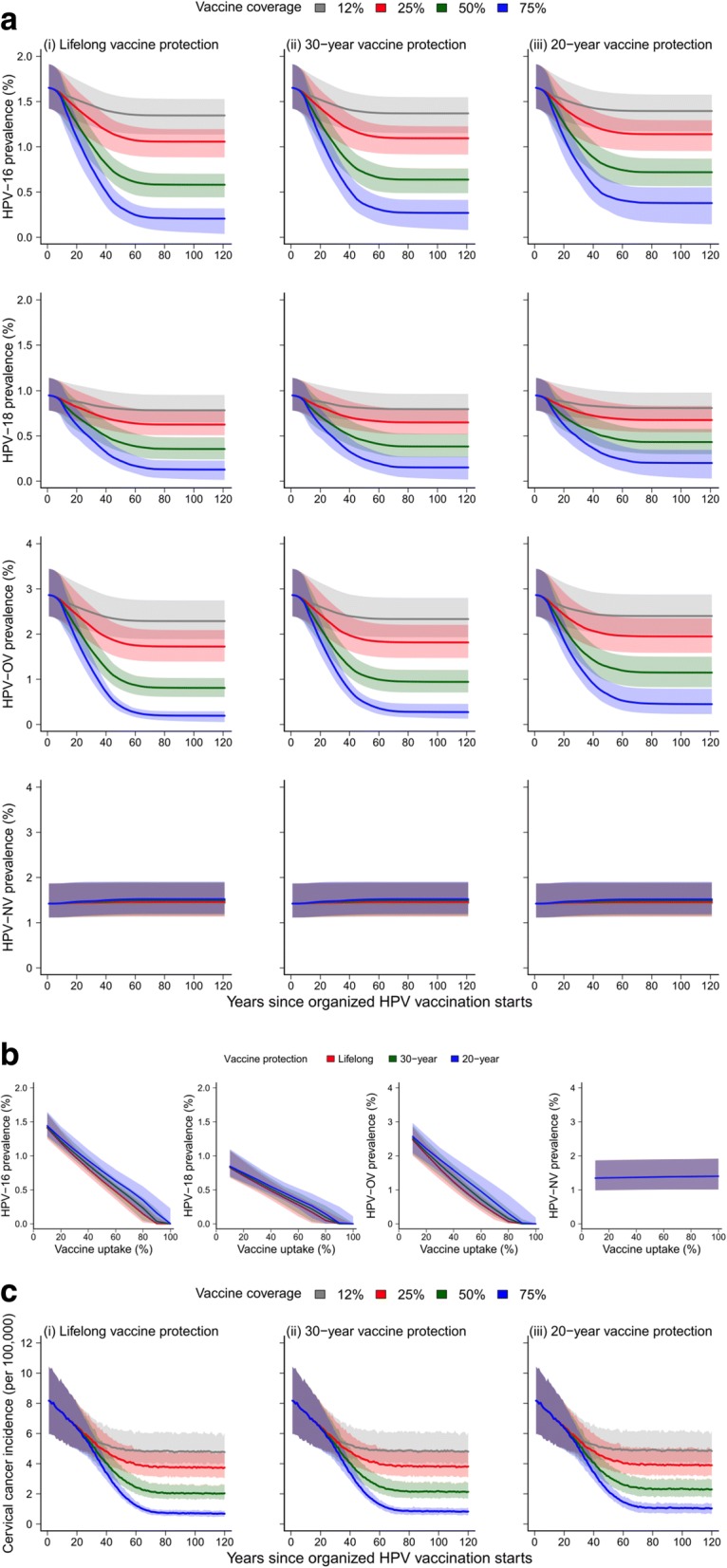
Epidemiologic impact of routine female adolescent HPV vaccination. The curves and shades indicate the medians and 95% central ranges of the outcomes across all 100 probabilistic sensitivity analysis scenarios on natural history parameters, respectively. **a** Age-standardized HPV prevalence over time. **b** Age-standardized HPV prevalence after 100 years of routine vaccination as a function of vaccine uptake. **c** Age-standardized incidence of cervical cancer over time

The prevalence of HPV-16, HPV-18, and HPV-OV decrease monotonically over time and reached a steady state 50–60 years after routine female vaccination began (Fig. 3a). This agrees with intuition because, in order for the routine vaccination program to confer maximal population-level benefit, all sexually active women in the population must have had the opportunity to receive the vaccine at age 12. Unsurprisingly, the prevalence of HPV-NV was constant over time because the nonavalent vaccine provides no protection against these HR-HPV types. Vaccine-type HR-HPV prevalence decreased with vaccine uptake as the latter increased from 10% to 90% with weak decreasing marginal return (Fig. 3b). Vaccine-type HR-HPV would have been eliminated if vaccine protection lasted for more than 30 years and routine vaccine uptake was higher than 90%. This result is consistent with the recent review of herd immunity threshold for HPV vaccination [6, 45]. The decrease in age-standardized incidence of cervical cancer during the first 20 years is not caused by vaccination but instead attributed to increased screening uptake in Hong Kong since CSP was launched in 2004 (Fig. 3c; see Additional file 1 for details of cervical screening in Hong Kong). Because HPV infections take at least 20 years to progress into malignancy in most cases of cervical cancer, the differential impact of vaccine uptake between status quo and routine vaccination on cervical cancer incidence would not be apparent until 20 years after routine vaccination had begun. Population-level benefit of routine vaccination on cancer incidence reached steady state 70–80 years after routine vaccination began. Compared to status quo opportunistic vaccination, routine vaccination with 25%, 50%, and 75% uptake further reduced the age-standardized cervical cancer incidence in year 100 by 21%, 57%, and 85%, respectively, with lifelong vaccine-induced protection, and by 19%, 51%, and 78%, respectively, with 20-year protection.

### Comparative threshold vaccine costs (TVC) between CBA and CEA

**Fig. 4:**
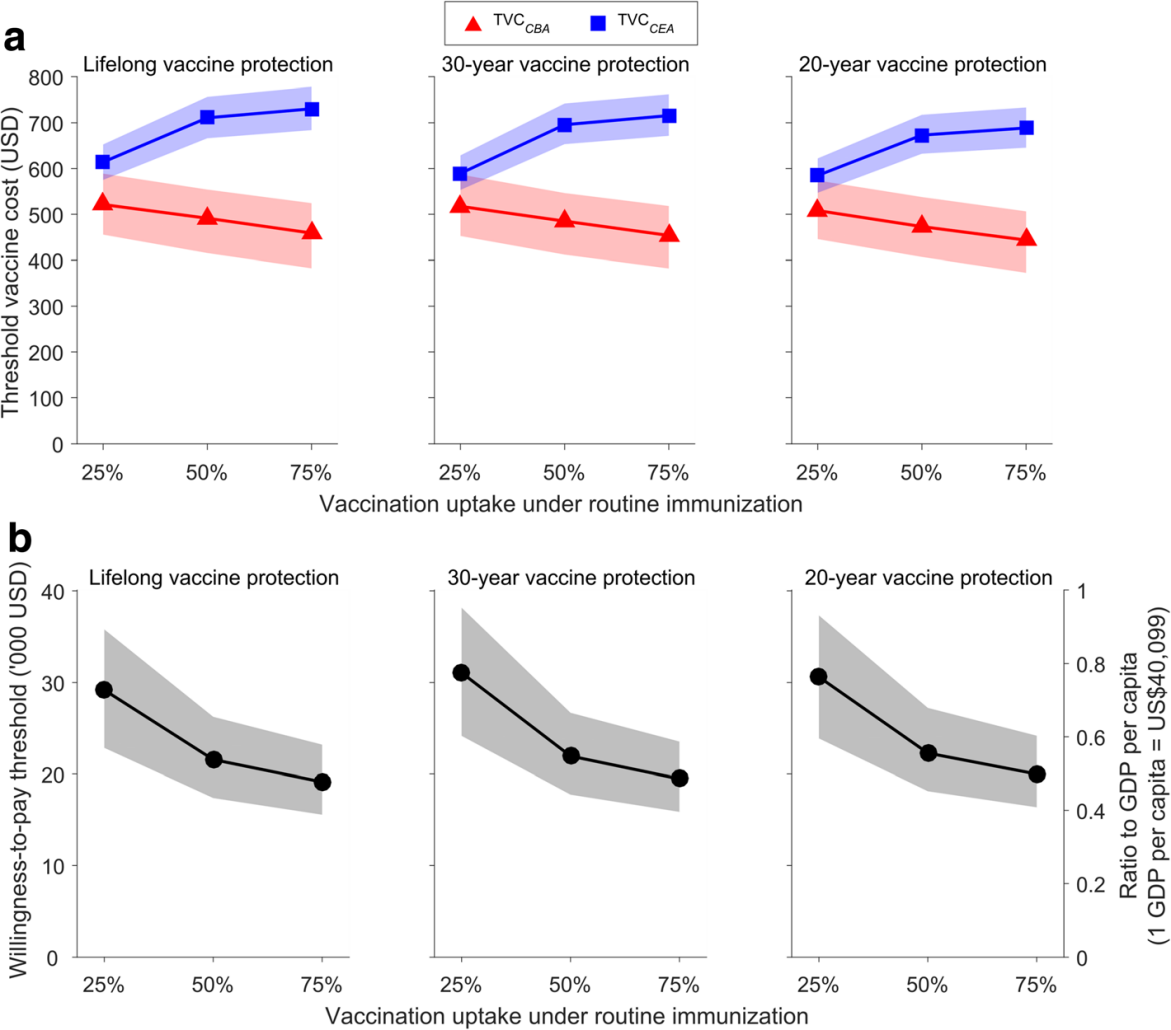
Comparative threshold vaccine cost between cost-benefit analysis and cost-effectiveness analysis. The curves and shades indicate the medians and 95% central ranges of the outcomes, respectively, across all 10,000 probabilistic sensitivity analysis combinations of natural history and health economic parameter values. The outcomes at 25%, 50%, and 75% vaccine uptake are used to estimate the outcomes at other vaccine uptake levels using linear interpolation. **a** Threshold vaccine cost, i.e., the maximum cost for vaccination at which routine vaccination of girls at age 12 is cost-beneficial (*TVC*_*CBA*_) and cost-effective (*TVC*_*CEA*_) compared to status quo vaccine uptake (12%) at the current market price (US$284 for the two-dose schedule). **b** The willingness-to-pay threshold at which *TVC*_*CBA*_ = *TVC*_*CEA*_. The GDP per capita in Hong Kong is US$40,099

As expected, the TVC decreased (i.e., became economically more stringent) as the duration of vaccine protection decreased (Fig. 4a). As vaccine uptake increased, *TVC*_*CBA*_ decreased but *TVC*_*CEA*_ increased with *TVC*_*CBA*_ > *TVC*_*CEA*_ across all scenarios considered: *TVC*_*CBA*_ was lower than *TVC*_*CEA*_ by approximately 13%, 30%, and 36% when vaccine uptake was 25%, 50%, and 75%, respectively (Fig. 4a). When the vaccine provided only 20 years of protection and vaccine uptake was 75% (i.e., the scenario under which *TVC*_*CBA*_ was the lowest), the total cost for fully vaccinating one girl would need to be less than US$444 ($373–506) and $689 ($646–734) for routine HPV vaccination to be cost-beneficial and cost-effective, respectively. Compared to this scenario, *TVC*_*CBA*_ (*TVC*_*CEA*_) increased by 2.3% (3.9%) if vaccine protection duration increased to 30 years or longer. If vaccine uptake decreased from 75% to 50%, *TVC*_*CBA*_ increased by 6.6% whereas *TVC*_*CEA*_ decreased by 2.3%. For *TVC*_*CBA*_ and *TVC*_*CEA*_ to be similar, the willingness-to-pay threshold would need to be approximately US$30,000 for vaccine uptake at 25% and US$20,000 for vaccine uptake at 75% (Fig. 4b).

## Discussion

### Main conclusions and their implications

We have evaluated the cost-benefit and cost-effectiveness of routine female adolescent nonavalent HPV vaccination for reducing cervical cancer burden in Hong Kong. Our results suggest that, at a vaccine uptake of between 25% and 75%, routine vaccination for 12-year-old girls (i.e., regardless of their sexual activity characteristics) as part of a centrally funded program represents good value for money if the cost of fully vaccinating one girl is no greater than US$444 ($373–506) and $689 ($646–734), respectively. The current market price of fully vaccinating one girl at age 12 at the FPAHK (as of November 2017) is $284, and the tender prices for bulk purchases in Italy, Norway, South Africa, and Spain were 66–77% lower than market prices [46]. Thus, we believe that the tender price of a centrally procured HPV vaccine in Hong Kong is likely to be well below the lower limit of our most conservative TVC estimate ($373) and thus provide the basis for a routine female adolescent HPV vaccination program that is both cost-beneficial and cost-effective.

Furthermore, because of the lack of local data, our analysis did not examine many of the benefits of HPV vaccination such as protection against anogenital warts, recurrent respiratory papillomatoses, or vulvar, vaginal, penile, anal, and oropharyngeal cancers. The value of protection against these non-cervical cancers has been estimated to be almost as great as the value of protecting against cervical cancers in some scenarios [47]. Further, our CBA is based on human capital calculations, valuing health in terms of a woman’s productive capacity, and does not capture the additional value that people put on averting suffering due to disease. If these considerations are taken into account, then the TVC for the CBA is likely to be even greater than that for the CEA. Therefore, it is almost certain that centrally funded routine HPV vaccination for 12-year-old girls will be both cost-beneficial and cost-effective in Hong Kong.

To our knowledge, our study is the first to compare the CBA and CEA implications of HPV vaccination. For Hong Kong, the CBA threshold vaccine cost is always lower (i.e., economically more stringent) than its CEA counterpart, i.e., *TVC*_*CBA*_/*TVC*_*CEA*_ < 1. The generalizability of this finding to other populations hinges on two other factors. The first factor is that CBAs in Hong Kong consider only changes in economic productivity (and not individual willingness-to-pay to avoid premature death as in the US or UK). Intuitively, *TVC*_*CBA*_/*TVC*_*CEA*_ increases with the ratio of average personal income to willingness-to-pay threshold. The second factor is the age distribution of cervical cancer in relation to retirement age and life expectancy. HPV vaccination will be (1) more cost-beneficial if the gap between the average age of cervical cancer cases and retirement age increases; and (2) more cost-effective if the gap between the average age of cervical cancer and life expectancy increases. Consider an illustrative comparison among Hong Kong, the US (with one GDP per capita as the willingness-to-pay threshold, i.e., approximately $58,000), and the UK (with £20,000–30,000 as the willingness-to-pay threshold). The ratio of average personal income to willingness-to-pay threshold is approximately 0.5 for Hong Kong, 0.72 for the US, and > 0.7 for the UK. On the other hand, the median age of cervical cancer diagnosis is 45 in the UK, 49 in the US, and 52 in Hong Kong, whereas the female life expectancy is 82, 79, and 87, respectively. The retirement age in these populations are similar. Applying the rationale above, the difference between *TVC*_*CBA*_ and *TVC*_*CEA*_ is likely to be significantly smaller for the US and UK compared to Hong Kong, and thus CBA and CEA will likely result in similar conclusions on the health economics of HPV vaccination in the US and UK.

### Comparison to other studies

A recently published review of cost-effectiveness studies of HPV vaccination [48], together with a more updated literature search, generated seven CEAs of nonavalent HPV vaccination in high-income countries [10, 49–54] and two for low- and middle-income countries [55, 56]. Most of these studies focused on switching from the use of either the bivalent or quadrivalent vaccines to nonavalent vaccine in existing vaccination programs [10, 49, 50, 52–54], while one study examined providing additional nonavalent vaccines to females who have already received three doses of quadrivalent vaccine [51]. Thus, they all differed from our study, which focused on comparing a scenario of an organized program using nonavalent vaccination at high coverage to an existing opportunistic program also using nonavalent vaccination.

Despite the different model scenarios, some results from previous studies can be compared with ours. We estimate that the female-only organized HPV vaccination with 75% vaccine uptake and lifelong vaccine protection would reduce cervical cancer incidence by 85% compared to 12% opportunistic vaccine uptake. In the CEAs for high-income countries, the additional reduction in cervical cancer for routine vaccination compared to no vaccination (which is the most similar scenario to the opportunistic vaccination scenario in our study) ranged between 70% and 92%, depending on vaccine uptake of females and males [10, 49, 50, 52, 54].

Although not explicitly stated, the TVC of nonavalent HPV vaccines for routine vaccination compared to no vaccination can be estimated from two previous studies where sufficient detail about the overall cost of vaccination is reported [52, 53]. In the Canadian CEA [53], the derived TVC for vaccination of 12-year-old females with a three-dose nonavalent vaccine schedule (as considered in the study) compared to no vaccination was estimated to be US$798. The estimation was based on a willingness-to-pay threshold of US$38,000 (CAD$40,000) per QALY gained, with a healthcare payer perspective, 80% female vaccination coverage, and 20-year duration of vaccine-induced protection. In another CEA for vaccinating both sexes in the US with a three-dose nonavalent vaccine schedule [52], the estimated TVC was US$959, using a willingness-to-pay threshold of US$50,000, a societal perspective, lifelong vaccine protection, and vaccine uptake of 46% and 29% among females and males aged 13–17 years, respectively. Given current opportunistic vaccine uptake of 12% [26] and assuming that the nonavalent vaccine is used for both organized and opportunistic vaccination, our study estimates that the TVC for organized female vaccination is US$689 at a willingness-to-pay threshold of US$40,099 per QALY gain, societal perspective, two-dose regimen, 75% vaccine coverage, and 20-year duration of vaccine-induced protection. The slightly lower TVC in our study is probably because (1) we did not consider non-cervical diseases in our study; (2) the opportunistic program that we considered already generates some herd protection so less benefit is expected from an organized program; and (3) we assumed only 20-year duration of vaccine protection.

### Strengths and limitations of the study

Our study has several other important limitations. First, we assumed that the duration and transmissibility of HPV infection are the same for males and females. While some evidence from other settings suggests that this is not generally true [9], Hong Kong does not have the necessary data (e.g., HPV prevalence or seroprevalence among males) for us to account for such heterogeneity. Second, we have not considered potential changes to cervical cancer screening (cytology is the most common primary screening method) and coverage after routine HPV vaccination begins. Depending on vaccine uptake, screening guidelines may be updated accordingly to optimize the cost-effectiveness and/or cost-benefit of screening [57]. Moreover, the use of primary HPV testing for cervical cancer screening will likely improve the positive predictive value of screening when the uptake of HPV vaccination is high [49]. Third, for model parsimony, we have assumed that assortativity in sexual mixing is the same for both sexes, which may not be accurate. Fourth, the health utility parameters in this study are based on studies from other settings that may not accurately reflect the situation in Hong Kong. Fifth, because there is no evidence on the societal willingness-to-pay threshold for cervical cancer in Hong Kong, we used one GDP per capita, which is the lowest willingness-to-pay threshold used by all the vaccination CEA studies in Hong Kong reviewed in Wong et al. [38]. The TVC from a CEA would be lower if the willingness-to-pay threshold is lower than that assumed here. Sixth, our CBA relies on valuing avoided morbidity and mortality using human capital calculations. The CBA threshold vaccine cost might be different if other methods are used (e.g., friction cost method [58] or approaches based on value of statistical life years [59]). Finally, the validity of the inferred parameters is limited by the data available for model parameterization and the associated assumptions imposed for fitting the model to these data. For example, transitions between CIN grades are based on data with 2-year follow-up periods and assumed to be Markovian, which might be inaccurate (e.g., we assume that a lesion is equally likely to clear regardless of how long it has persisted within the same CIN1 or CIN2 grade).

A major strength of our study is that, as we evaluate the health economics of HPV vaccination, we simultaneously characterize sexual mixing in Hong Kong by fitting the transmission model to epidemiological data [60]. The resulting parameter estimates suggest that sexual mixing in Hong Kong is, as would be anticipated, highly assortative by both age and sexual activity level. The level of age assortativity inferred in our study is comparable to that reported in sexual surveys from the UK and Australia, which lends support to the validity of our estimates. Given the substantial costs of sexual surveys and the difficulty of eliciting truthful responses on sexual behaviors, inference of sexual mixing parameters from epidemiologic data of sexually transmitted diseases (including HPV, chlamydia, etc.) is a potentially fruitful but underused methodology for understanding sexual behaviors in the population. Our study provides a first step in this direction.

The Greater Bay Area (GBA) Initiative in the 13^th^ 5-year plan (2016–2020) of China aims to link the cities of Hong Kong, Macau, Guangzhou, Shenzhen, Zhuhai, Foshan, Zhongshan, Dongguan, Huizhou, Jiangmen, and Zhaoqing into an integrated economic, business, and technology hub that constitutes an area of 56,000 km^2^, a combined population of 68 million, and an economy of $1.51 trillion. Given the low uptake of HPV vaccination and cervical cancer screening and the high burden of cervical cancer in these cities, prevention of cervical cancer will likely be a top-priority public health issue in the GBA Initiative. Over the next decade, demographics, sexual mixing, and disease transmission in these heterogeneous cities will be substantially impacted by the massive increase in short- and long-term human mobility and interaction brought about by the GBA Initiative. A recent study showed that interstate migration has a strong impact on the population-level benefit of HPV vaccination in the US because of herd immunity and the long duration between HPV infection and resultant cervical cancer [61]. As such, the GBA cities will need to coordinate their evaluations and policies to maximize the benefit of their HPV vaccination programs. Our study for Hong Kong provides a robust basis for the development of such a cooperative framework.

## Conclusions

Routine HPV vaccination of 12-year-old females is highly likely to be cost-beneficial and cost-effective in Hong Kong. Inference of sexual mixing parameters from epidemiologic data of prevalent sexually transmitted diseases (i.e., HPV, chlamydia, etc.) is a potentially fruitful but largely untapped methodology for understanding sexual behaviors in the population.

## Additional file

Additional file 1: **Table S1.** The distribution of individuals with no, low, and high level of sexual activity in each age group. **Table S2.** Posterior distributions of inferred parameters. **Table S3a.** Probability distributions of cervical cytology testing. **Table S3b.** Probability of cytology results given true health states. **Table S4.** Probability distributions of cost parameters. **Table S5.** Probability distribution of QALY weights for different health outcomes. **Figure S1.** Schematic of the natural history model for HR-HPV infection and cervical cancer among females. **Figure S2.** Trace plots and the posterior distributions of the fitted parameters. (PDF 544 kb)

## Abbreviations

CBA: cost-benefit analysis
CEA: cost-effectiveness analysis
CIN: cervical intraepithelial neoplasia
CSP: cervical screening programme
FPAHK: The Family Planning Association of Hong Kong
GBA: Greater Bay Area
GDP: gross domestic product
HPV: human papillomavirus
HPV-NV: HPV non-vaccine HR-HPV
HPV-OV: HPV other vaccine types
HR-HPV: high-risk HPV
TVC: threshold vaccine cost
