# Supplementary material for: Simultaneously characterizing the comparative economics of routine female adolescent nonavalent human papillomavirus (HPV) vaccination and assortativity of sexual mixing in Hong Kong Chinese: a modeling analysis
Source: BMC Med. 2018 Aug 17;16:127. doi: 10.1186/s12916-018-1118-3 (PMC6097427; doi:10.1186/s12916-018-1118-3)
Supplement: Supplementary file 1 — Table S1. The distribution of individuals with no, low, and high level of sexual activity in each age group. Table S2. Posterior distributions of inferred parameters. Table S3a. Probability distributions of cervical cytology testing. Table S3b. Probability of cytology results given true health states. Table S4. Probability distributions of cost parameters. Table S5. Probability distribution of QALY weights for different health outcomes. Figure S1. Schematic of the natural history model for HR-HPV infection and cervical cancer among females. Figure S2. Trace plots and the posterior distributions of the fitted parameters. (PDF 544 kb) [file 12916_2018_1118_MOESM1_ESM.pdf]

Supplementary

Simultaneously characterizing the comparative economics of routine female adolescent nonavalent human papillomavirus (HPV) vaccination and assortativity of sexual mixing in Hong Kong Chinese: a modeling analysis

Horace CW Choi[1,2,3†], Mark Jit[1,4,5], Gabriel M Leung[1], Kwok-Leung Tsui[2], Joseph T Wu[1†]

[1] WHO Collaborating Centre for Infectious Disease Epidemiology and Control, School of Public Health, Li Ka Shing Faculty of Medicine, The University of Hong Kong, Hong Kong

[2]Department of Systems Engineering and Engineering Management, City University of Hong Kong, Hong Kong

[3]Department of Clinical Oncology, Li Ka Shing Faculty of Medicine, The University of Hong Kong, Hong Kong

[4]Modelling and Economics Unit, Public Health England, United Kingdom

[5]Department of Infectious Disease Epidemiology, London School of Hygiene and Tropical Medicine, London, United Kingdom

† These authors contributed equally

Sexual mixing

We use the survey data published by the FPAHK (http://www.famplan.org.hk) to construct the sexual activity matrix (Table S1) as follows:

1. Individuals age below 10 or above 69 are assumed to be sexually inactive.
2. The sexual activity distributions for individuals age 13-14 (text in red) are based on the data for Forms 1-2 students in The Report of Youth Sexuality Study 2011.
3. The sexual activity distributions for individuals age 10-12 (text in green) are linearly interpolated from the distributions in steps 1 and 2.
4. The sexual activity distributions for individuals age 15-19 (text in purple) are based on the data in Section Form 3- Form 7 in The Report of Youth Sexuality Study 2006.
5. The sexual activity distributions for individuals age 20-24 (text in magenta) are based on the data in Section Aged 18-27 Youths in The Report of Youth Sexuality Study 2006.
6. The sexual activity distributions for males age 30-69 (text in blue) are based on Table 7.5a in the 2001 Men's Health Survey.
7. The sexual activity distributions for females age 30-69 (text in orange) are extrapolated from the distribution for females age 20-24 (from step 5) assuming that the age effect on the distribution of low and high levels of sexual activity for females is the same as that for males (from step 6).
8. The data in both The Report of Youth Sexuality Study 2006 and Men's Health Survey 2001 suggest that those with a high level of sexual activity had an average of 2.5 sexual partners during the past 6 months.

	Sexual activity level (no. of sexual partners during the 6 months)					
	Male			Female		
Age (years)	None (0)	Low (1)	High (>1)	None (0)	Low (1)	High (>1)
10-12	0.993	0.007	0.000	0.998	0.002	0.000
13-14	0.985	0.015	0.000	0.995	0.005	0.000
15-19	0.900	0.062	0.038	0.945	0.046	0.009
20-24	0.580	0.303	0.117	0.632	0.291	0.077
25-29	0.286	0.579	0.135	0.312	0.556	0.132
30-39	0.102	0.798	0.100	0.111	0.766	0.122
40-49	0.094	0.827	0.079	0.102	0.794	0.103
50-59	0.099	0.849	0.052	0.108	0.815	0.077
60-69	0.362	0.604	0.034	0.394	0.580	0.025

Table S1. The distribution of individuals with no, low and high level of sexual activity in each age group. Individuals with one and multiple sexual partners during the past 6 months are regarded as having low and high sexual activity levels, respectively.

Adjusted contact rates

For the transmission model to be internally consistent, the following balance rule must be satisfied at all times:

$$c_{f,a,u}\rho_{f,a,u,b,v}(t)N_{f,a,u}(t)=c_{m,b,v}\rho_{m,b,v,a,u}(t)N_{m,b,v}(t)$$

$$c_{f,a,u}\rho_{f,a,u,b,v}(t)N_{f,a,u}(t)=c_{m,b,v}\rho_{m,b,v,a,u}(t)N_{m,b,v}(t)$$

This balance rule simply states the fact that the number of sexual partnerships that females from stratum (f,a,u) form with males from stratum (m,b,v) is the same as the number of sexual partnerships that males from stratum (m,b,v) form with females from stratum (f,a,u). At any given time *t*, the degree to which the balance rule is violated could be measured by:

$$D_{a,u,b,v}(t)=\frac{c_{f,a,u}\rho_{f,a,u,b,v}(t)N_{f,a,u}(t)}{c_{m,b,v}\rho_{m,b,v,a,u}(t)N_{m,b,v}(t)}.$$

To ensure that the balance rule is satisfied at all times, the adjusted contact rates are:

$$c^{*}_{f,a,u,b,v}(t)=c_{f,a,u}\left(D_{a,u,b,v}(t)\right)^{-(1-\theta)}$$
$$c^{*}_{m,b,v,a,u}(t)=c_{m,b,v}\left(D_{a,u,b,v}(t)\right)^{\theta}$$

Following common practice, we choose $\theta=0.5$ which means that the relevant parameters of females and males are adjusted to the same degree.[1]

Natural history

Figure S1 shows the natural history of HR-HPV infection and cervical cancer among females in the model. Individuals enter the population without HPV infection at birth and become sexually active as early as age 10. For females who are infected with HPV, the infection could progress to precancerous states (CIN1, CIN2 and CIN3). We assume that individuals with CIN3 would not recover naturally. Disease progression rates and clearance rates are assumed to depend on HPV type but not age.

Local cervical cancer without symptoms may become symptomatic or progress to more advanced stages of cervical cancer without symptoms. In the absence of screening, cervical cancer is diagnosed only when symptoms develop in which the patient is immediately treated. Females with symptomatic cervical cancer are subjected to stage-specific (local, regional or distant) probability of cancer-associated death.[2] We assume that recovery from cervical cancer does not confer natural immunity against reinfection. The progression rates of cervical cancer and the cervical cancer-related death rates are assumed to be independent of age, sexual activity level and HPV type.[3]

Basic compartmental epidemic models based on ordinary differential equations (ODEs) assume that the duration of each compartment is exponentially distributed.[4] For any given mean duration, the probability that the duration is shorter than the mean is higher in exponential distribution than in more biologically plausible distributions such as Erlang and lognormal distributions. Such a difference would have little effect for disease states milder than CIN3 because their durations are relatively short compared to cervical screening intervals. However, given that the expected duration of CIN3 is much longer (>10 years on

average),[5] assuming that the duration of CIN3 is exponentially distribution might artificially lower the effectiveness of cervical screening. As such, we assume that the time from CIN3 to asymptomatic local cervical cancer is an Erlang-4 distribution.[4,6] This multiple-compartment component is important for the stochastic cohort simulation model which simulates the impact of cervical screening on prevention of cervical cancer. We refer to a local study for cervical cancer specific survival by the International Federation of Gynaecologists and Oncologists (FIGO) staging system.[7] The reported 5-year survival rates are 90.9%, 71.0%, 41.7% and 7.8% for FIGO stage I, II, III and IV, respectively. Cancer patients who remain alive 5 years after cancer diagnosis are regarded as cancer survivors.[8] In the model, cancer survivors are moved to the health state “No infection (susceptible)” and are susceptible to new HPV infection.

We assume that the transmission, progression and regression parameters for HPV infections in males are the same as that in females.[3] We do not consider HPV-associated diseases among males.[3,9]

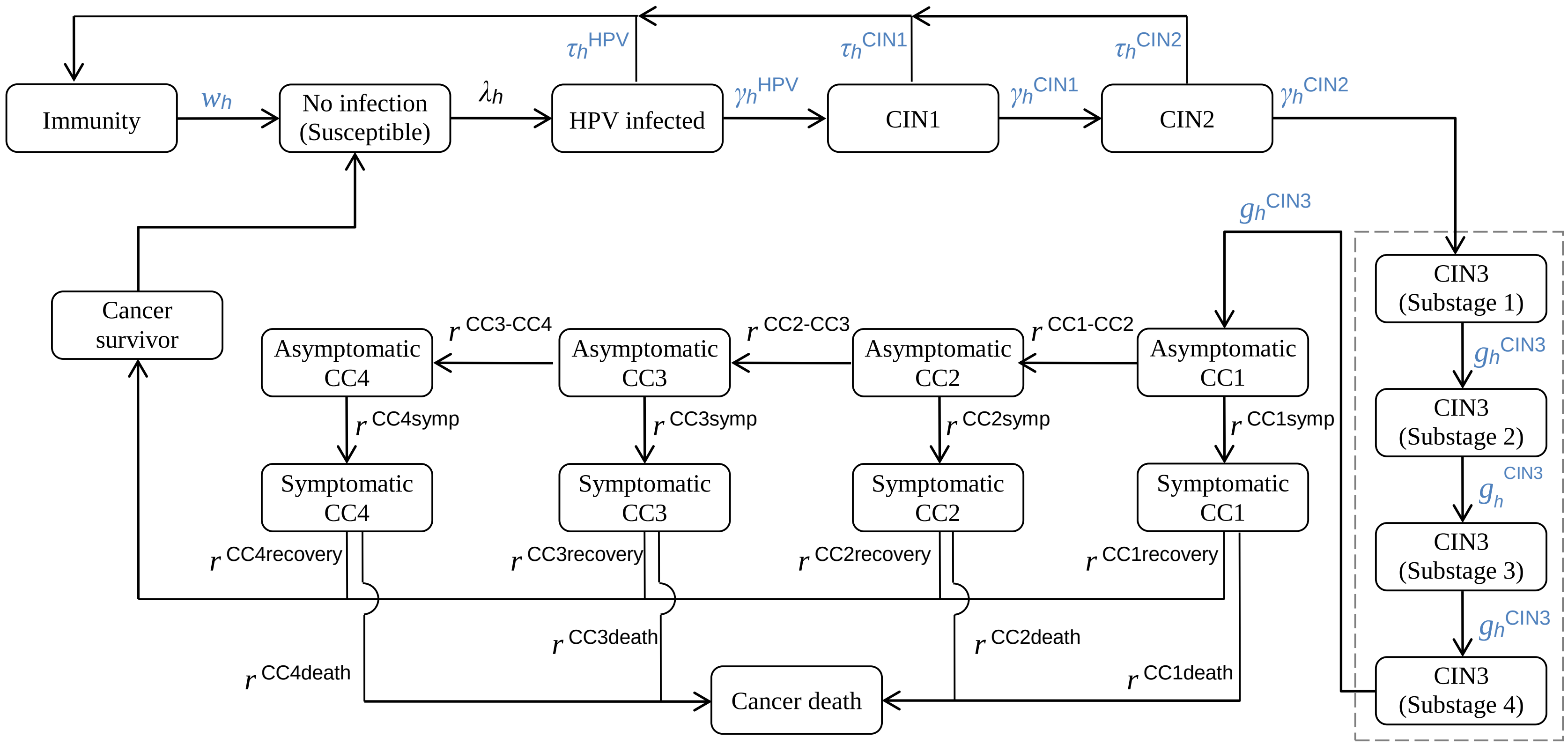

Figure S1. Schematic of the natural history model for HR-HPV infection and cervical cancer among females. Abbreviations: CC1 to CC4, cervical cancer in International Federation of Gynecology and Obstetrics (FIGO) stage I to IV correspondingly. λ_h is the force of infection (FOI) for HPV class h. γ_h^X and τ_h^X are the progression and clearance rate for disease state X with HPV class h. w_h is the waning rate of natural immunity against HPV class h. The progression of CIN3 to asymptomatic CC1 is assumed to follow an Erlang-4 distribution with mean $1/\gamma_h^{CIN3}$, i.e. $\gamma_h^{CIN3} = g_h^{CIN3}/4$.

Model parameterization

We estimate the model parameter values using the Metropolis-Hasting algorithm with non-informative flat priors for all parameters[10,11] (see Table S2). When formulating the likelihood function for model fitting, we assume:

1. The data on age-specific HPV prevalence[12] and proportion of HPV types in cervical cancer cases[13] follow binomial distributions;
2. The data on age-specific cervical cancer incidence[14] follow Poisson distributions;
3. The data on disease progression and clearance (for different stages of HPV infection)[15,16] follow multinomial distributions.

The trace plot and Geweke diagnostic[17] indicate that the MCMC chain converges (Figure S2). As such, we estimate the posterior distribution by running the Metropolis-Hasting algorithm for 300,000 iterations with a burn-in of 150,000 iterations without thinning. Table S2 shows the summary statistics of the posterior distributions.

(A) Inferred parameters on natural history		Posterior median (95% CrI)			
Parameter	Description	HPV-16	HPV-18	HPV-OV	HPV-NV
β_h	Transmission probability per sexual partnership	0.75 (0.50, 0.96)	0.88 (0.60, 0.98)	0.93 (0.80, 0.99)	0.61 (0.50, 0.71)
$1/\gamma_h^{HPV}$	Mean duration: progression from HPV infection to CIN1	8.7 (7.2, 11.3)	5.9 (4.3, 8.6)	10.7 (8.9, 12.8)	11.2 (9.4, 13.5)
$1/\gamma_h^{CIN1}$	Mean duration: progression from CIN1 to CIN2	3.9 (2.7, 5.3)	3.4 (2.3, 5.2)	2.7 (2.1, 3.9)	
$1/\gamma_h^{CIN2}$	Mean duration: progression from CIN2 to CIN3	4.2 (3.0, 6.4)	4.2 (2.9, 6.7)	4.5 (2.9, 7.3)	
$1/\gamma_h^{CIN3}$	Mean duration: progression from CIN3 to cervical cancer	22 (18, 28)	22 (16, 30)	32 (20, 40)	
$1/\tau_h^{HPV}$	Mean duration: clearance of HPV infection	2.2 (1.9, 2.5)	1.4 (1.2, 1.7)	1.6 (1.5, 1.8)	1.6 (1.5, 1.7)
$1/\tau_h^{CIN1}$	Mean duration: clearance of CIN1	3.2 (2.1, 4.8)	3.0 (2.2, 4.3)	1.3 (1.1, 1.7)	
$1/\tau_h^{CIN2}$	Mean duration: clearance of CIN2	3.2 (2.5, 4.3)	3.2 (2.3, 4.4)	1.7 (1.4, 2.4)	
$1/w_h$	Mean duration of natural immunity	15.9 (2.7, 83.2)	16.9 (3.6, 74.8)	0.68 (0.51, 1.69)	0 (assumed)

(B) Inferred parameters on sexual mixing		Posterior median (95% CrI)
W_1	Age (yrs) at which susceptibility and infectiousness begin to fall	21 (16, 25)
W_2	Age (yrs) at which susceptibility and infectiousness stop falling and begin to plateau	24 (21, 27)
M	Relative transmission probability for individuals older than W_1	0.47 (0.41, 0.53)
ε_A	Degree of assortativeness for sexual mixing across ages	0.77 (0.29, 0.97)
ε_S	Degree of assortativeness for sexual mixing across sexual activity levels	0.98 (0.89, 0.99)
σ_g	Spread of age preference (yrs) in forming sexual partnership	2.05 (0.21, 4.90)

Table S2. Posterior distributions of inferred parameters.

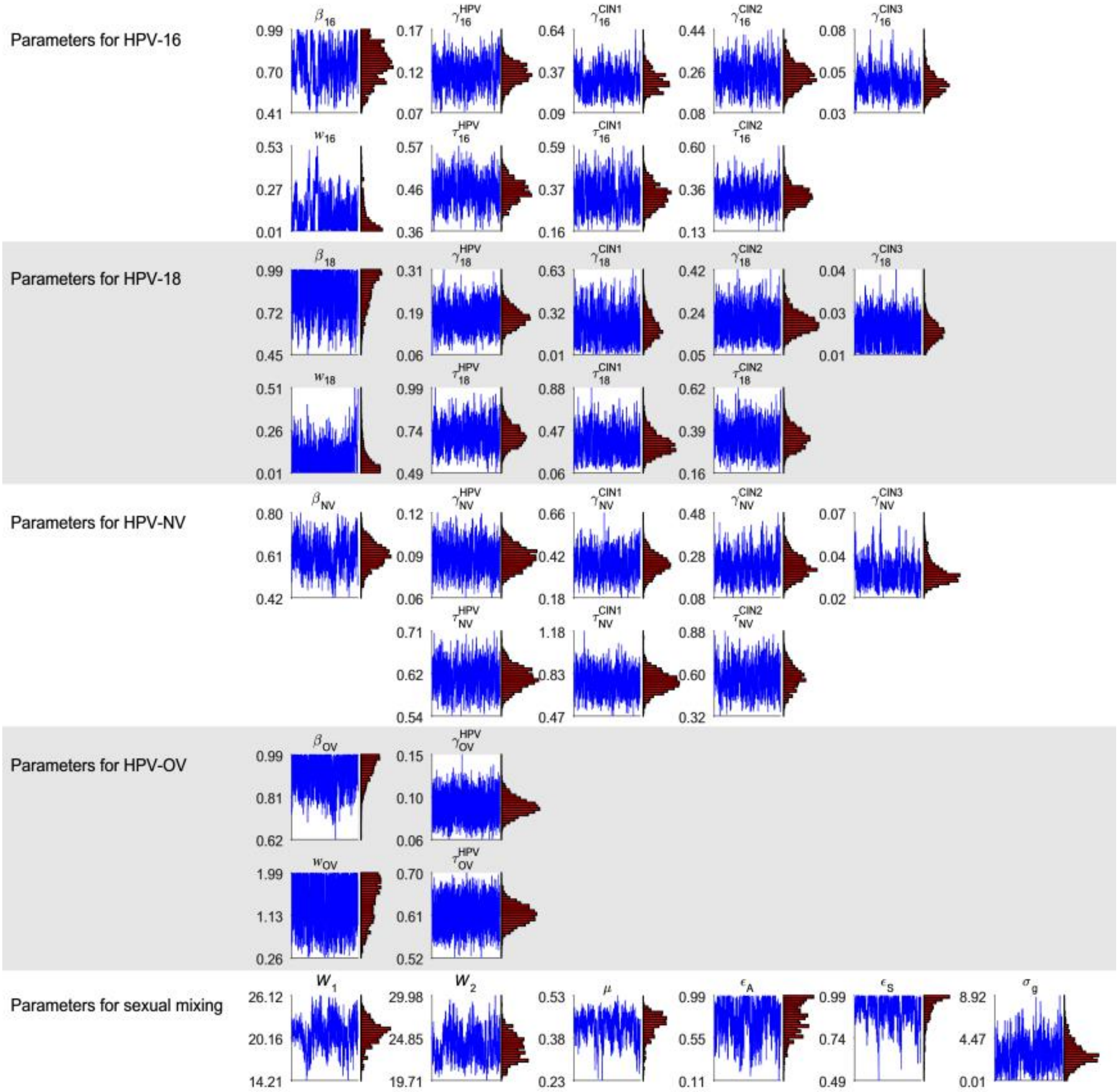

Figure S2. Trace plots and the posterior distributions of the fitted parameters.

Cervical screening

The Cervical Screening Programme (CSP) in Hong Kong was launched in 2004.[18] CSP recommends eligible women aged 25-64 to adopt 1-,1-,3-yearly cervical cytology screening, i.e. screening annually for their first two years of screening and then triennially if their screening results remain negative. The latest statistics from the Department of Health suggest that 70% of eligible women have had undergone screening at least once, 60% of whom have had their most recent screening episode during the last 3 years.[18] This finding is consistent with our previous survey in 2009.[19] As such, we make the following assumptions regarding the uptake of cervical cancer screening after CSP has launched in 2004:

i. 70% of females begin screening when they reach age 25;
ii. 60% of the females in (i) will then follow the 1-,1-,3-yearly screening;
iii. The remaining 40% of (i) will attend screening annually in the first 2 years and then every 75 months afterwards.[19]

We assume the following screening uptake before the launch of CSP [19]:

i. 40% of females begin screening when they reach age 25 years since 1980 when cervical screening was introduced into systematic antenatal care in Hong Kong;[19,20]
ii. 60% of the females in (i) attend screening regularly at 1- to 3-year interval;[19]
iii. The screening uptake increases linearly from 40% in 1980 to 70% in 2004 when CSP was launched.[18]

Table S3 summarizes (a) sensitivity and specificity of cervical cytology and (b) the probability of cytology results given the true health states assumed in our analysis.[21-23] Furthermore, we set the sensitivity of colposcopy to CIN1 and CIN2/3 lesions at 81%, based

on the findings from the two cervical screening trials in China.[24,25] We assume same sensitivity to CIN lesions as suggested by another overseas study.[26] We set the sensitivity of colposcopy to cervical cancer at 100%. We also set 100% sensitivity of biopsy to CIN lesions and cervical cancers.

**Test characteristic for cervical cytology**[21]	**Distribution**	**References**
Sensitivity		
CIN1	T(0.70, 0.50. 1.00)	[21]
CIN2/3	T(0.80, 0.50. 1.00)	[21]
Cervical cancer	T(1.00, 0.95, 1.00)	Assumed
Specificity	T(0.95, 0.90. 1.00)	[21]

Table S3a. Probability distributions of cervical cytology testing. $T(a,b,c)$ denotes triangular distribution that ranges from b to c with mode a.

**Probability of cytology results**				
	True health states			
Cytology results	Normal	CIN1	CIN2/3	Cancer
ASCUS	0.704	0.517	0.380	0.286
LSIL	0.269	0.448	0.450	0.286
HSIL	0.027	0.035	0.170	0.428

Table S3b. Probability of cytology results given true health states. Abbreviations: ASCUS, atypical squamous cells of undetermined significance; CIN, cervical intraepithelial neoplasia; HSIL, high-grade squamous intraepithelial lesion; LSIL, low-grade squamous intraepithelial lesion. Ref.: [21-23].

Cost parameters

We estimate that the costs for screening and treatments based on the private charges in the public healthcare system (released by the Department of Health and listed in the 2013 Gazette[27]) which accounts for approximately 90% of hospitalization in Hong Kong.[28] Table S4 summarizes the probability distributions of the costs used in our analysis. The underlying assumptions are provided below.

The costs of screening comprise:

1. The cost of cytology Pap smear for routine screening;
2. The cost of colposcopy for the follow-up of low-grade squamous intraepithelial lesion (LSIL) and high-grade squamous intraepithelial lesion (HSIL) after abnormal Pap smear.
3. Time and transportation cost for women to go to clinics for screening, where the time cost is based on the average income of females by age groups.[29,30]

We estimate the treatment costs for CIN2/3 and cervical cancer based on public hospital charges for private patients with standard treatments (i.e. assuming that the public hospitals make these charges just for covering their costs instead of making profits).[27] The private charges for in-patients include the fees for general nursing, core pathology investigations, catering and also domestic services.[31] The treatment cost of cervical cancer comprises the cost of hospitalization for receiving Wertheim's hysterectomy, brachytherapy, and overnight infusion chemotherapy. We assume 67.4% of the women who die from cervical cancer would receive palliative care with a mean hospitalization duration of 42.5 days.[32]

**Cost**	**Distribution (USD)**	**References**
Cytology test	N(67.9, 17)	[27]
Colposcopy + biopsy	N(779, 195)	[27]
Treatment for CIN2 or CIN3<br>Loop electro-surgical excision procedure (LEEP)	N(1869, 467)	[27]
Treatment for local cervical cancer<br>Wertheim's hysterectomy	N(13,914, 3,479)	[27]
Treatment for regional cervical cancer<br>Radiotherapy + chemotherapy + brachytherapy	N(31,051, 7,763)	[27]
Treatment for distant cervical cancer<br>Palliative radiotherapy + palliative chemotherapy	N(23,476, 5,869)	[27]
Palliative care (per day)	T(601, 479, 723)	[27,32]
Time cost (half day)	N(30.1, 7.5)	[33]
Transportation	N(6.4, 1.6)	[29]

Table S4. Probability distributions of cost parameters. $N(a,b)$ denotes normal distribution with mean a and standard deviation b. $T(a,b,c)$ denotes triangular distribution that ranges from b to c with mode a.

Cost-benefit analysis

We use the human capital approach to estimate the productivity loss incurred due to (i) screening, (ii) treatment for cervical intraepithelial lesions and cervical cancer, and (iii) premature death from cervical cancer.[34] These productivity losses comprise (i) loss of economic income that would have been earned by the individual and (ii) loss of workplace productivity (for the individual's employer) that would have been averted if the individual has not experienced these events.[35] Nicholson et al. (2006) estimated that the absence of an employee would generate an extra loss of 61% times the employee's income to the employer and thus to the entire society, i.e. a wage multiplier of 1.61.[35] When estimating productivity loss for different age groups, we adjust for the age-specific unemployment and labor force participation rate.[36] Specifically, given age group *i*, let w_i be the average daily income for employed persons, r_{ui} be the unemployment rate, r_{fi} be the labor force participation rate, and m_w be the wage multiplier. The adjusted potential daily productivity loss from a female in that age group *i* is $w_i m_w r_{fi}(1-r_{ui})$.

We make the following assumptions regarding the duration of productivity loss:

1. Each episode of screening is associated with 2 days of absence from work: 1 day for attending screening and 1 day for reviewing the screening result.
2. Each episode of CIN2/3 treatment is associated with 3 days of absence from work: 1 day for receiving the treatment and 2 days for resting.
3. Treatment of cervical cancer is associated with 6 months of absence from work for local, regional and distant stage.[8]
4. Premature death from cervical cancer is associated with absence from work between the time of death and the average retirement age which we assume to be 65 years.[33]

Cost-effectiveness analysis

When calculating quality-adjusted life-years (QALYs), we rely on health utility parameters from overseas HPV vaccination cost-effectiveness studies (Table S5) because analogous data are not available in Hong Kong.[21,37,38] QALY is estimated by summing women's health utilities incurred throughout their lifetimes. We assume that there is no health utility loss for undiagnosed or asymptomatic cervical lesions and cervical cancer.[39] Health utility is reduced (i) upon abnormal screening results; (ii) upon diagnosis and treatment (if any) of CIN; and (iii) during treatment of cervical cancer. Furthermore, we assume that cancer survivors have lower health utilities for their first 5 years after recovery.[8]

There is no consensus on willingness-to-pay threshold below which health interventions are deemed cost-effective in Hong Kong. As such, we use GDP per capita (approximately US$40,099 for Hong Kong during 2012-2016[40]), as the societal willingness-to-pay threshold.

**Health utility**	**Distribution**	**References**
Screening	N(0.98, 0.005)	[38]
ASCUS	N(0.94, 0.015)	[38]
Normal colposcopy result	N(0.95, 0.013)	[38]
CIN1	N(0.81, 0.022)	[38]
CIN2/3	N(0.87, 0.033)	[38]
Local cervical cancer	T(0.65, 0.49, 0.81)	[41]
Regional cervical cancer	T(0.56, 0.42, 0.70)	[41]
Distant cervical cancer	T(0.48, 0.36, 0.60)	[41]
Cancer survivor	T(0.62–0.97, 0.47–0.73, 0.78–0.99)	[41]

Table S5. Probability distribution of QALY weights for different health outcomes. *N*(*a*,*b*) denotes normal distribution with mean *a* and standard deviation *b*. *T*(*a*,*b*,*c*) denotes triangular distribution that ranges from *b* to *c* with mode *a*. Standard deviations for health utilities were based on the corresponding utility detriments from perfect health (i.e., 1) and an assumption of 0.25 coefficient of variation (CV).[42]

References

1. Barnabas RV, Laukkanen P, Koskela P, Kontula O, Lehtinen M, and Garnett GP. Epidemiology of HPV 16 and cervical cancer in Finland and the potential impact of vaccination: Mathematical modelling analyses. *PLoS Medicine* 2006; **3**(5):e138.
2. Goldie SJ, Grima D, Kohli M, Wright TC, Weinstein MC, and Franco EL. A comprehensive natural history model of HPV infection and cervical cancer to estimate the clinical impact of a prophylactic HPV-16/18 vaccine. *International Journal of Cancer* 2003; **106**:896-904.
3. Kim JJ, and Goldie SJ. Health and economic implications of HPV vaccination in the United States. *New England Journal of Medicine* 2008; **259**(8):821-832.
4. Wearing HJ, Rohani P, and Keeling MJ. Appropriate models for the management of infectious diseases. *PLoS Medicine* 2005; **2**(7):e174.
5. Schiffman M, Castle PE, Jeronimo J, Rodriguez AC, and Wacholder S. Human papillomavirus and cervical cancer. *The Lancet* 2007; **370**(9590):890-907.
6. Gunther OP, Ogilvie G, Naus M, Young E, Patrick DM, Dobson S, et al. Protecting the next generation: what is the role of the duration of human papillomavirus vaccine-related immunity? *Journal of Infectious Diseases* 2008; **197**(12):1653-1661.
7. Cheung FY, Mang OWK, and Law SCK. A population-based analysis of incidence, mortality, and stage-specific survival of cervical cancer patients in Hong Kong: 1997-2006. *Hong Kong Medical Journal* 2011; **17**(2):89-95.
8. de Kok IMCM, van Ballegooijen M, and Habbema JDF. Cost-effectiveness analysis of human papillomavirus vaccination in the Netherlands. *Journal of the National Cancer Institute* 2009; **101**(15):1083-1092.
9. Johnson HC, Elfstrom KM, and Edmunds WJ. Inference of type-specific HPV transmissibility, progression and clearance rates: A mathematical modelling approach. *PLoS ONE* 2012; **7**(11):e49614. doi:10.1371/journal.pone.0049614.
10. Korostil IA, Peters GW, Cornebise J, and Regan DG. Adaptive Markov Chain Monte Carlo forward simulation for statistical analysis in epidemic modelling of human papillomavirus. *Stat Med* 2013; **32**(11):1917-1953.
11. Calderhead B. A general construction for parallelizing Metropolis-Hastings algorithms. *Proc Natl Acad Sci USA* 2014; **111**(49):17408-17413.
12. Chan PKS, Ho WCS, Wong MCS, Chang AR, Chor JSY, and Yu M-Y. Epidemiologic risk profile of infection with different groups of human papillomaviruses. *Journal of Medical Virology* 2009; **81**:1635-1644.
13. Chan PKS, Ho WCS, Yu M-Y, Pong W-M, Chan ACL, Chan AKC, et al. Distribution of human papillomavirus types in cervical cancers in Hong Kong: current situation and changes over the last decades. *International Journal of Cancer* 2009; **125**(7):1671-1677.
14. Hong Kong Cancer Registry. 2016. Available at: http://www3.ha.org.hk/cancereg/. Accessed on 3 March 2016.
15. Insinga RP, Perez G, Wheeler CM, Koutsky LA, Garland SM, Leodolter S, et al. Incident cervical HPV infections in young women: transition probabilities for CIN and infection clearance. *Cancer Epidemiology, Biomarkers & Prevention* 2011; **20**(2):287-296.
16. Moscicki A-B, Ma Y, Wibbelsman C, Darragh TM, Powers A, Farhat S, et al. Rate of and risks for regression of cervical intraepithelial neoplasia 2 in adolescents and young women. *Obstetrics and Gynecology* 2010; **116**:1373-1380.

17. Geweke J. Evaluating the accuracy of sampling-based approaches to the calculation of posterior moments, in *Bayesian Statistics*, J.M. Bernardo, J.O. Berger, A.P. Dawid *et al.*, eds. 1992, Oxford University Press: Oxford. p. 169-193.
18. Cervical Screening Programme, Department of Health. 2016. Available at: http://www.cervicalscreening.gov.hk/eindex.php. Accessed on 6 January 2016.
19. Wu JT. Cervical cancer prevention through cytologic and human papillomavirus DNA screening in Hong Kong Chinese women. *Hong Kong Med J* 2011; **17**(Suppl 3):S20-S24.
20. Leung GM, Woo PPS, McGhee SM, Cheung ANY, Fan S, Mang O, et al. Age-period-cohort analysis of cervical cancer incidence in Hong Kong from 1972 to 2001 using maximum likelihood and Bayesian methods. *Journal of Epidemiology and Community Health* 2006; **60**:712-720.
21. Kim JJ, Wright TC, and Goldie SJ. Cost-effectiveness of alternative triage strategies for atypical squamous cells of undetermined significance. *JAMA* 2002; **287**(18):2382-2390.
22. Goldie SJ, Kim JJ, and Wright TC. Cost-effectiveness of human papillomavirus DNA testing for cervical cancer screening in women aged 30 years or more. *Obstetrics and Gynecology* 2004; **103**(4):619-631.
23. Goldhaber-Fiebert JD, Stout NK, Ortendahl JD, Goldie SJ, and Salomon JA. Modeling human papillomavirus and cervical cancer in the United States for analyses of screening and vaccination. *Population Health Metrics* 2007; **5**:11.
24. Belinson J, Qiao YL, Pretorius R, Zhang WH, Elson P, Li L, et al. Shanxi Province Cervical Cancer Screening Study: a cross-sectional comparative trial of multiple techniques to detect cervical neoplasia. *Gynecol Oncol* 2001; **83**(2):439-444.
25. Qiao Y-l, Sellors JW, Eder PS, Bao Y-p, Lim JM, Zhao F-h, et al. A new HPV-DNA test for cervical-cancer screening in developing regions: a cross-sectional study of clinical accuracy in rural China. *Lancet Oncol* 2008; **9**(10):929-936.
26. Da Forno PD, Holbrook MR, Nunns D, and Shaw PAV. Long-term follow-up of patients following negative colposcopy: a new gold standard and its implications for cervical screening. *Cytopathology* 2003; **14**(5):281-286.
27. Hospital Authority. *Revisions to List of charges: Gazette No. 12/2013*. 2013.
28. Hospital Authority. *Hospital Authority Statistical Report*. 2013. Available at: http://www.ha.org.hk/gallery/ha_publications.asp?Library_ID=15&lang=en. Accessed on 14 February 2013.
29. Woo PPS, Kim JJ, and Leung GM. What is the most cost-effective population-based cancer screening program for Chinese woman? *Journal of Clinical Oncology* 2007; **25**(6):617-624.
30. Census and Statistics Department. *Women and Men in Hong Kong Key Statistics (2017 edition)*. 2017.
31. Hospital Authority. *Fees and Charges*. n.d. Available at: http://www.ha.org.hk/haho/ho/cs/v3/serviceguide_feenchg-en.htm. Accessed on 22 January 2013.
32. Tse DMW, Chan KS, Lam WM, Leu KS, and Lam PT. The impact of palliative care on cancer deaths in Hong Kong: a retrospective study of 494 cancer deaths. *Palliat Med* 2007; **21**(3):425-433.
33. Census and Statistics Department. 2017. Available at: http://www.censtatd.gov.hk/home/index.jsp. Accessed on 19 April 2017.
34. Hanly P, Timmons A, Walsh PM, and Sharp L. Breast and prostate cancer productivity costs: A comparison of the human capital approach and the friction cost approach. *Value Health* 2012; **15**(3):429-436.

35. Nicholson S, Pauly MV, Polsky D, Sharda C, Szrek H, and Berger ML. Measuring the effects of work loss on productivity with team production. *Health Econ* 2006; **15**(2):111-123.
36. Census and Statistics Department. *Women and Men in Hong Kong Key Statistics 2015 edition*. 2015.
37. Goldie SJ, Kohli M, Grima D, Weinstein MC, Wright TC, Bosch FX, et al. Projected clinical benefits and cost-effectiveness of a humam papillomavirus 16/18 vaccine. *Journal of the National Cancer Institute* 2004; **96**(8):604-615.
38. Insinga RP, Glass AG, Myers ER, and Rush BB. Abnormal outcomes following cervical cancer screening: event duration and health utility loss. *Medical Decision Making* 2007; **27**(4):414-422.
39. Elbasha EH, Dasbach EJ, and Insinga RP. Model for assessing human papillomavirus vaccination strategies. *Emerging Infectious Diseases* 2007; **13**(1):28-41.
40. Census and Statistics Department. *Table 30: Gross domestic product (GDP), implict price deflator of GDP and per capita GDP*. 2016. Available at: http://www.censtatd.gov.hk/hkstat/sub/sp250.jsp?subjectID=250&tableID=030&ID=0&productType=8. Accessed on 3 February 2017.
41. Myers ER, Green S, and Lipkus I. *Patient preferences for health states related to HPV infection: visual analog scales vs time trade-off elicitation* at *21st International Papillomavirus Conference and Clinical Workshop*. 2004. Mexico City, Mexico.
42. Jit M, Chapman R, Hughes O, and Choi YH. Comparing bivalent and quadrivalent human papillomavirus vaccines: economic evaluation based on transmission model. *Br Med J* 2011; **343**:d5775.
